# For Me, Quality of Life Is When I Get Through the Day Without Thinking: I Won't Make It: A Participatory Qualitative Study of Health‐Related Quality of Life in Rare Liver Diseases

**DOI:** 10.1111/hex.70721

**Published:** 2026-06-14

**Authors:** Anouck Alary, Jane Sattoe, Lynda Sifer, Isabelle Aujoulat, José Mira, Guenda Bernegger, Giada Danesi, Eva Gil‐Hernández, Pura Ballester, Mercedes Guilabert, Doortje Nipius, Jeanina Kousemaker‐Nieuwdorp, Kay van der Meer‐Kuiper, Stefania Cannavò, Cecile Drossart, Liliane Veniat, María Caiata Zufferey, Agnes Dumas

**Affiliations:** ^1^ INSERM, Aix‐Marseille Univ, IRD, ISSPAM, SESSTIM (Economic and Social Sciences of Health and Medical Information Processing), CALIPSO team Marseille France; ^2^ Rotterdam University of Applied Sciences, Research Centre Innovations in Care Rotterdam The Netherlands; ^3^ UCLouvain, Institute of Health & Society, Academic Centre for health promotion‐RESO Brussels Belgium; ^4^ Miguel Hernandez University of Elche, Department of Health Psychology Elche Spain; ^5^ FISABIO, Alicante‐Sant Joan Healthcare District Alicante Spain; ^6^ SUPSI (University of Applied Sciences and Arts in Southern Switzerland), DEASS (Department of Business Economics, Health and Social Care) CPPS (Competence Centre for Healthcare Practices and Policies) Manno Switzerland; ^7^ FISABIO, Atenea Research Group Sant Joan d'Alacant Spain; ^8^ UCAM (Universidad Catolica de Murcia), Facultad de Farmacia y Nutricion Murcia Spain

## Abstract

**Background:**

Vascular liver diseases (VLDs) predominantly affect young adults and require lifelong monitoring. The health‐related quality of life (HRQoL) of patients with VLDs is deteriorated, with high levels of fatigue and depressive symptoms, but their lived experience and unmet needs remain unknown. To fully integrate the patients’ perspective and generate actionable insights, we conducted a qualitative peer‐research study.

**Methods:**

Twelve peer researchers conducted interviews with patients in France, Spain, Switzerland, and the Netherlands. Analysis proceeded in three phases: individual inductive thematic analysis independently made by peer and academic researchers, followed by an international workshop to build a shared thematic framework. Third, directed content analysis, informed by the shared framework, was made by an academic researcher.

**Results:**

From 27 interviews, five themes were identified, mapping the disease's impact on quality of life from diagnosis onward, and throughout the patient journey. First, the diagnosis trajectory was characterised by ‘uncertainty and biographical disruption’. Then, long‐term symptoms were described as impacting daily life through the experience of ‘chronic uncertainty, loss of ability and social exclusion’. Participants’ accounts also reflected the social impact of VLDs, which resulted in ‘renegotiating roles, ties and identities’. All along the care journey, their healthcare experience could be marked by ‘relational and institutional gaps shaping illness experience’. Finally, ‘the sense of belonging to a community of peers’ was highlighted as a potential but lacking resource.

**Conclusions:**

Living with a rare VLD involves uncertainty, unpredictable fatigue, stigma, and social disruption, alongside efforts to maintain dignity and autonomy. While patients can demonstrate resilience, this does not negate ongoing needs. Improving quality of life requires addressing not only symptoms such as fatigue, anxiety or sexual dysfunction but requires guidance on physical activity, nutrition or occupational and financial challenges.

**Patient and Public Contribution:**

Ten patients and two caregivers were involved as peer researchers throughout the study, ensuring that priorities, interpretations, and conclusions reflected patient‐defined concerns. All peer researchers contributed to the design, data collection and analysis activities. Due to professional and health constraints, about half of the PRs could only participate in the first local analysis phase. PRs were financially compensated.

## 1 Introduction

With advances in diagnosis and treatment, survival has improved for many rare diseases [1], shifting attention toward the quality of life of patients living with these conditions [2]. Given the early age at diagnosis and the chronic nature of many rare diseases, multiple life domains may be affected, including education, work, income, personal relationships, family life, or social life. However, in rare or chronic liver diseases, most of the literature describing the quality of life of patients continues to focus on disease‐related variables, using standardised multidimensional scales designed to capture functional and medical outcomes. These tools often overlook patients’ subjective well‐being, psychological adaptation, and social coping mechanisms, thereby reproducing a narrow biomedical perspective that fails to integrate a patient‐centred view, notably for patients living with rare diseases [2, 3].

Rare vascular liver diseases (VLDs) are a typical example of rare diseases affecting young people, for which diagnosis has improved dramatically in recent decades. VLDs are characterised by a primary obstruction of the hepatic veins (Budd‐Chiari syndrome or BCS) or the portal venous (non‐cirrhotic portal vein thrombosis or PVT), leading to portal hypertension and potential liver dysfunction. These conditions are rare (BCS: ~1/million; PVT: ~1/100,000) [4, 5], with BCS being less common but often more severe, as up to 50% of BCS patients have a myeloproliferative neoplasm. Genetic factors may contribute to both diseases [5, 6], which can also occur in children. On average, patients are young as compared to other chronic diseases: the median age at diagnosis of PVT is around 55 years, while patients with BCS are younger, with a median age at diagnosis between 35 and 40 years old. Prognosis has significantly improved over the last decades, but complications, notably gastrointestinal bleeding, refractory ascites, severe bacterial infections, hepatic encephalopathy, remain serious without proper management, requiring long‐term treatment, monitoring, and sometimes invasive procedures. Pregnancy poses additional risks for women of childbearing age (50% of BCS, 20% of PVT), such as recurrent thrombosis, bleeding, miscarriage, preterm birth and stillbirth [7].

Despite numerous calls for clarification, the terms ‘quality of life’ and ‘health‐related quality of life’ HRQL are often used interchangeably, although, quality of life refers to a global construct of well‐being, encompassing all aspects of a person's life, whereas HRQoL represents a narrower subset that specifically captures the impact of health status on well‐being [8]. Prior work assessing the HRQoL of patients with VLDs has shown that fatigue and depressive symptoms were common, especially among women, those with comorbidities, financial difficulties, or patients who experienced hepatic encephalopathy [9]. Yet, such tools incompletely capture what patients themselves may identify as most meaningful: autonomy and self‐determination in managing their condition, social participation (work, family life), recognition of their illness by healthcare providers, employers, and peers, as well as the preservation of identity, normalcy and financial security [10, 11, 12, 13].

This study aimed to understand how individuals living with VLDs perceived their overall quality of life and well‐being, and to identify previously unexplored unmet needs, with the goal of providing recommendations improving care and support. Given the limited existing empirical evidence, in order to fully integrate the patients’ perspective into knowledge creation and dissemination, we conducted a qualitative peer‐research study to generate actionable insights for clinical practice, which consisted in a partnership with patients, caregivers and academic researchers at each step of the research.

## 2 Materials and Methods

### 2.1 Design and Setting

This study was a participatory qualitative study in which patients and caregivers acted as peer researchers (PRs) and were involved at all stages of the study. This study was carried out in the framework of a European project, building upon existing collaborations between patient organisations, clinical and research teams. Conducted in 2024‐2025 in France (FR), Spain (SP), Switzerland (CH) and the Netherlands (NL), it included 12 PRs (three per country), all patients with VLDs or other rare liver diseases, or caregivers, working with support from 10 academic researchers from diverse disciplines, experts in qualitative health research. The study was grounded in a broad and flexible theoretical understanding of health‐related quality of life [14], while being informed by sociological approaches to the experience of chronic illness [10, 11].

### 2.2 Recruitment and Training of PRs

PRs were contacted through patients’ organisations (FR, SP and NL) or through clinicians involved in the project (CH). A budget was secured for PRs in each country: PRs could choose to directly receive the funding or decide that their organisation would receive the funding. Training sessions were organised in each country to familiarise PRs with the conduct of qualitative interviews and thematic analysis following a common training support elaborated by two of the research teams (CH and FR). Training was planned as a 2‐day face‐to‐face workshop combining formal presentations, interview simulations (individual and group role plays), and collective feedback sessions, alongside informal moments fostering collaboration within teams. The training covered the rationale and objectives of the project, principles and techniques of qualitative interviewing (e.g., empathy, active listening, open‐ended and follow‐up questions), ethical and regulatory aspects (including informed consent and general data protection regulation [GDPR] requirements), and practical guidance for preparing, conducting, and analysing interviews. The workshop also included the collaborative development of interview guides and guidance on the use of a structured analysis sheet following each interview.

### 2.3 Recruitment of Participants

Participants in the qualitative study (interviewees) were contacted through various ways: through a questionnaire study conducted within the same project (FR), with the help of clinicians partnering the project, during clinical visits (SP and CH) and/or through a call for participation from a patients’ organisation (NL). All the participants were diagnosed either with BCS or PVT and were aged > 18 years old.

### 2.4 Data Collection

Data collection consisted of semi‐structured interviews conducted by PRs with patients living with PVT or BCS. ARs arranged the meetings between PRs and interviewees, except in Spain, where the research nurse facilitated the connection, allowing to thoroughly inform research participants about the aim and the peer‐research design of the study. During the data collection phase, multiple individual and collective debriefings were organised between PRs and ARs to provide logistic and scientific support to PRs and share preliminary findings. Interviews were audio‐recorded with the participants’ consent to ensure accurate data capture and then transcribed verbatim for analysis.

### 2.5 Design of the Interview Guides

Each PR team designed its own interview guide to preserve the participatory nature of the project, ensure cultural relevance, and support PR ownership, while sharing core domains to enable cross‐country analysis. Guides were developed through collective brainstorming led by PRs, with ARs supporting the structuring of questions and suggesting optional topics. All four guides covered diagnosis experience, treatment, symptoms and their impact, emotions and fears, coping strategies, life changes, effects on personal and social life, unmet needs and expectations. Some topics varied by country: income impact was not addressed in Switzerland, while quality of life was explored more broadly in the Netherlands, including positive aspects of the impact of illness.

### 2.6 Data Analysis

Given the international and multidisciplinary composition of the research team, and the involvement of patients and caregivers not familiar with qualitative analysis, we opted for a staged approach with three key phases. In the first phase, inductive analysis was conducted by each local team using rapid analysis techniques. These techniques are diverse [15], and those used here consisted in the creation of summary templates capturing structural attributes (e.g., case‐specific context) and main themes with codes and illustrating quotes. Each interview template was completed independently by both an AR and the PR conducting the interview, including field notes. All PRs read all interviews and were invited to complete a short template on interviews made by others. Themes and overarching patterns were then discussed between PRs and ARs in local workshops, and each local team provided a thematic framework.

In the second phase, these frameworks were discussed in an international workshop gathering PRs and ARs. After this workshop, frameworks were compiled into a broader international framework using a cumulative approach, and agreed following further meetings. In the third phase, data collected in local languages during the interviews were translated into English by bilingual research team members, with AI‐assisted support in two countries. A directed content analysis was conducted by an AR (AA), informed by the international framework while also allowing for additional themes not initially included [16]. Excel sheets were used to manage data and frameworks, and ensure transparency and interoperability [17]. A research report was written in English, including exemplifying quotes of each country and then back‐translated, and reviewed by PRs and ARs for feedback, verification, and refinement. ARs then summarised key findings, focusing on shared results across teams while acknowledging country‐specificities. They interpreted data within existing literature and discussed the draft with PRs. Due to professional and health constraints, about half of the PRs could only participate in the first phase of the analysis, especially younger and more professionally active participants, particularly men. This article reports results following the COREQ checklist for transparent reporting.

### 2.7 Ethics

Ethical approval was obtained in each participating country. Participant confidentiality was ensured through pseudonymization procedures, and data were stored and transferred using secure procedures compliant with national regulations and the GDPR. Given the everyday ethical challenges of participatory research [18], measures were implemented to ensure PR well‐being and voluntary participation. ARs regularly reminded PRs that they could withdraw at any time. During data collection, analysis sheets included a self‐observation section on expectations, concerns, and willingness to continue. ARs also organised individual and group debriefings before and after interviews, maintained open communication channels, and created messaging groups (e.g., WhatsApp) to support ongoing contact between PRs and ARs.

## 3 Results

Of the 12 PRs engaged in the study, 7 were women, 10 were patients while 2 were caregivers, and 7 had a prior experience of interviewing, whether through their work experience or through PAO activities. The 12 PRs conducted 25 interviews (up to three per PR), averaging 50 min (range 30–90). Two additional interviews in Switzerland were conducted by ARs due to language barriers (total 27). Most interviews were face‐to‐face, in participants’ home. Table 1 shows participants’ characteristics: half were women, mean age ~50, two‐thirds diagnosed with PVT, nearly half diagnosed in their 30s, a third in their 40s or later, with a mean 12 years since diagnosis (range 0–40). Pseudonyms and rounded ages were used to protect privacy.

**Table 1:** Characteristics of the 27 participants.

Characteristics	*N* (%)
Country	9 (33%) France; 8 (30%) Spain; 6 (22.2%) Switzerland; 4 (15%) Netherlands
Sex	15 women (56%); 12 men (44%)
Age	7 (26%) in their 30s–40s; 14 (52%) in their 50s; 6 (22%) in their 60s or older
Diagnosis^a^	19 (70%) PVT; 8 (30%) BCS
Age at diagnosis	5 (18%) in their 20s or younger; 12 (44%) in their 30 s; 10 (37%) in their 40s or older
Employment status	19 (70%) employed; 5 (18%) inactive or unemployed; 2 (7%) on disability pension; 1 (4%) on sick leave
Setting of the interview	17 (63%) face to face; 6 (22%) online; 4 (15%) by phone

^a^ Many participants were also living with comorbid conditions in addition to their rare VLD diagnosis. For reasons of confidentiality, these associated conditions cannot be specified. Altogether, 13 (48%) participants were concerned with blood or immune disorders (*n* = 6), history of stroke (*n* = 2), other liver diseases (*n* = 3), previous injuries or accidents (*n* = 2).

Five overarching themes were identified, detailing the disease's impact during the diagnosis phase and in subsequent stages of the patient's journey, including follow‐up and long‐term adaptation. First, the diagnosis trajectory was characterised by ‘uncertainty and biographical disruption’. Then, long‐term symptoms were described as impacting daily life through the experience of ‘chronic uncertainty, loss of ability and social exclusion’. Participants’ accounts also reflected the social impact of VLDs, which resulted in ‘renegotiating roles, ties and identities’. All along the care journey, their healthcare experience could be marked by ‘relational and institutional gaps shaping illness experience’. Finally, ‘the sense of belonging to a community of peers’ was highlighted as a potential but lacking resource (Table 2).

**Table 2:** Thematic analytical framework: Five overarching themes, subthemes, interview frequency, and countries.

Overarching themes	Subthemes	Number of interviews	Countries
1. Diagnosis: Uncertainty and biographical disruption			
Diagnosis trajectory	Diagnostic uncertainty: Misdiagnosis or delayed diagnosis	13	FR, SP, NL, CH
	Uncertainty/fear	7	FR, SP, CH, NL
	Relief at diagnosis	2	NL, CH
	Experience of stigma	4	FR, SP
	Misdiagnosis (mistrust, resentment)	8	FR, SP, NL, CH
	Self‐advocacy	3	FR, NL, CH
Biographical disruption	Profound disruption of personal and professional lives	12	FR, SP, NL, CH
	Fear of imminent death, breakdown of previous life and professional plans	11	FR, SP, NL, CH
2. Long‐term symptoms and daily living: chronic uncertainty, loss of ability and feelings of social exclusion			
Physical long‐lasting symptoms and treatments affecting daily life	Unpredictable fatigue	17	FR, SP, NL, CH
	Abdominal discomfort, dietary adjustments and social withdrawal	8	FR, SP, NL, CH
	Altered body image affecting social confidence	5	FR, NL, CH
	Impaired sexual quality of life	1	FR
Long‐term psychological impact	Symptoms of anxiety and depression	13	FR, SP, NL, CH
	Reappraisal of the present moment and resilience	11	FR, SP, NL, CH
Caution, anticipation and vigilance in everyday life	Alcohol abstinence	7	FR, SP, NL
	Dietary restrictions	8	FR, SP, NL, CH
	Travel limitations	11	FR, SP, CH
	Fear of doing sport	11	FR, SP, NL, CH
	Motivation for doing sport	7	FR, SP, NL, CH
	Daily coping strategies to manage symptoms or reduce the burden of disease monitoring	5	FR, SP, NL, CH
	Mental cost of anticipation	4	FR, SP, NL
3. Renegotiating roles, ties and identities			
Relationships with close and extended circles	Inner circle: Gratitude and guilt about burdening relatives	4	FR, SP, NL, CH
	Extended circle: Disbelief and social withdrawal	6	FR, SP, NL
Personal life: disrupted life projects and expected roles	Reshaping reproductive choices	4	FR, SP, NL, CH
	Diminished capacity to fulfil expected parental roles	12	FR, NL
	Challenging couple stability	4	FR, SP, NL
Professional trajectories	Feeling limited in one's career	8	FR, SP, NL, CH
	Fear/experience of discrimination at work	7	FR, SP, NL
The cost of illness	Social cost of the disease	7	FR, SP, NL
4. Healthcare experience: from relational to institutional gaps			
Relational gaps	Banalization of symptoms/treatment side effects	8	FR, CH, SP
	Importance of good communication	2	NL, CH
Systemic gaps	Lack of care coordination among HCPs	7	SP, NL, CH
	Caregivers’ needs	9	FR, SP, NL, CH
5. The sense of belonging to a community of peers			
Peer community and mutual support	Sharing advice and limiting isolation	8	SP, NL, CH

### 3.1 The Diagnosis Trajectory: Uncertainty and Biographical Disruption

The diagnostic trajectory unfolded in two main phases. For many patients, it began with a prolonged period of diagnostic uncertainty characterised by repeated consultations, limited explanations, and stigmatising interactions. During this phase, symptoms were frequently minimised, misattributed or framed as consequences of lifestyle factors (e.g., alcohol use, obesity, stress), delaying appropriate investigations and undermining patients’ confidence in medical professionals. Several participants described having to engage in strong self‐advocacy, actively searching for specialised care in the absence of clear institutional guidance, sometimes leading to lasting resentment toward earlier medical encounters. As one participant explained: 'Since it's liver‐related, the most common question I got was whether I was an alcoholic. They asked me so many times that I started to doubt myself. I thought, 'Should I just say 'yes' so that they'll stop asking?'' (P8, woman in her 40s).

For others, this initial phase was shorter or absent, and diagnosis emerged through an abrupt and destabilising health crisis marked by acute decompensation, emergency hospitalisation, and severe complications. Interventions such as repeated paracenteses for ascites were described as physically exhausting and psychologically distressing: ‘They removed eight liters twice a week… it was hell’ (P12, woman in her 60s). These hospitalisations disrupted employment, family roles, and future plans, and were often narrated as moments of existential rupture. As one participant recalled: ‘I was 30, had my whole life ahead of me, and I saw my future slipping away’ (P5, woman in her 40s).

Whether preceded by a long period of uncertainty or occurring through a sudden medical crisis, diagnosis marked a transition into a second phase characterised by biographical disruption. The illness was no longer perceived solely as a set of symptoms, but as a condition reshaping identities, daily practices, and expectations for the future. Receiving a diagnosis could initially bring partial relief—especially when cancer or cirrhosis were ruled out: ‘Things became clearer after diagnosis… okay, everything is fine’ (P19, woman in her 30s). However, patients soon became aware of the long‐term implications of a chronic liver disease, initiating a process of identity redefinition and ongoing adaptation to a fundamentally altered life trajectory.

### 3.2 Long‐Term Symptoms and Daily Living: Chronic Uncertainty, Loss of Ability and Feelings of Social Exclusion

Following the acute phase, most participants gradually resumed daily routines; however, recovery was widely described as fragile and unstable. Rather than a clear return to ‘normal life,’ patients reported living with persistent symptoms requiring constant adaptation and anticipation.

#### 3.2.1 Physical Long‐Lasting Symptoms and Treatments Affecting Daily Life

##### 3.2.1.1 Unpredictable and Debilitating Fatigue

Fatigue—both physical and cognitive—was described as one of the most pervasive and disabling long‐term symptoms, particularly among participants who had BCS, and even more in those who had experienced hepatic encephalopathy. Its unpredictability was often more disruptive than its intensity, forcing patients to carefully plan activities and conserve energy: ‘Maybe, tomorrow, I will wake up, and, for the whole day, I will do the bare minimum: breakfast and then go back to bed' (P16, man in his 40s diagnosed with BCS). For some, quality of life was defined in negative terms, as the absence of collapse rather than the presence of vitality: ‘For me, quality of life is when I get through the day without thinking: I won't make it’ (P24, man in his 50s with an experience of hepatic encephalopathy).

##### 3.2.1.2 Abdominal Discomfort, Dietary Adjustments and Social Withdrawal

Digestive symptoms were also reported, including chronic diarrhoea, flatulence, and food intolerances, which required constant dietary adjustments and often interfered with social life. As one participant put it: ‘Nowadays, the most complicated thing is managing my digestion’ (P10, man in his 50s). Several participants felt insufficiently supported by HCPs in that regard and learned to manage symptoms through trial and error. To avoid embarrassment or discomfort, some limited eating outside the home, which contributed to social withdrawal and feelings of isolation. Besides, alcohol avoidance carried social costs, resulting in a sense of exclusion from rituals of sociability, especially in younger patients: ‘I find it harder to socialise, I go out less, I've become a bit more withdrawn’ (P11, woman in her 30s).

##### 3.2.1.3 Altered Body Image Affecting Social Confidence

Changes in physical appearance were another source of distress, particularly for women who had experienced ascites or large weight fluctuations, even years after. These bodily transformations reinforced a sense of bodily fragility and loss of control over one's appearance, affecting self‐image and social confidence: ‘I was really in a state of total collapse, with the lower half of my body looking like I was twelve months pregnant… I was completely deformed like Quasimodo!’ (P12).

##### 3.2.1.4 Impaired Sexual Quality of Life

Sexual health was discussed as a sensitive but significant dimension of well‐being. Treatments and physical symptoms affected libido and performance, sometimes leading patients to modify or discontinue medications, as a patient discontinuing betablockers explained ‘It disturbed our couple's life’ (P10).

#### 3.2.2 Long‐Term Psychological Impact

Beyond persistent physical symptoms, participants described substantial psychological distress following the acute phase of illness, describing caution and vigilance in everyday life due to the burden of reduced mobility and disease monitoring, and, for some, chronic anxiety or depression related to the fear of complications. Yet, several participants described processes of reappraisal and resilience.

##### 3.2.2.1 Symptoms of Anxiety and Depression

Anxiety was described as an enduring feature of life, often linked to fear of relapse, sudden deterioration, or life‐threatening complications. Even when medically stable, some reported a persistent fear and a sense of existential fragility, especially patients diagnosed with a BCS: ‘Even though I feel good, I know I can suddenly flip’ (P26, man in his 50s). This fostered emotional fatigue and hypervigilance, with patients closely monitoring bodily sensations and interpreting minor symptoms as potential warning signs.

##### 3.2.2.2 Caution, Anticipation and Vigilance in Everyday Life

Physical activity and mobility were constrained by medication side effects. Fear of injury or bleeding curtailed physical activities once taken for granted for patients treated with anticoagulants. Travel required careful planning, because of medications, planning for emergencies, and taking redundant precautions. Yet some participants deliberately chose to test their limits in order to preserve a sense of autonomy and continuity in the face of chronic illness: ‘Yesterday I tried rollerblading for a bit. My family was like: ‘What are you thinking? Be careful.’ I just want to try… to see what I can still do’ (P27, woman in her 30s).

Patients described daily coping strategies to manage symptoms or reduce the burden of disease monitoring. For those requiring regular anticoagulation monitoring, the use of a home self‐monitoring device was particularly valued, as it allowed them to check their INR (a standard parameter of blood coagulation) levels at home and avoid weekly trips to the laboratory for blood tests. Overall, rather than returning straightforwardly to ‘normal life’ after the acute phase of illness, patients described the need to adapt to what some called a ‘medicalized life’—a daily existence shaped by vigilance, planning, and self‐monitoring, which came with the mental cost of anticipation (for meals, travel, sport, exams…): ‘I have this form of caution… a more medicalized life, a slightly more alert life, but that doesn't prevent me from having a normal life.’ (P11).

Feelings of isolation were also common, partly due to reduced social participation and difficulties in sharing the ongoing psychological burden with relatives who perceived the crisis as over. Some participants reported depressive symptoms, loss of motivation and diminished projections into the future, particularly among patients with BCS,those who had experienced severe complications such as hepatic encephalopathy or repeated hospitalisations.

##### 3.2.2.3 Reappraisal of the Present Moment and Resilience

At the same time, psychological adjustment did not take a single form. Alongside anxiety and emotional exhaustion, several participants described processes of reappraisal and resilience, characterised by a reorientation toward the present and a recalibration of life priorities. Rather than long‐term planning, some adopted flexible temporal horizons and focused on immediate well‐being: ‘I don't plan too rigidly, because I know things can change. Enjoy now, don't postpone’ (P26). Illness was described as a biographical turning point that prompted reflection on previous lifestyles: ‘It put everything in perspective. I had always worked hard, long hours, travelled a lot, ate badly. Since my illness, I try to take life more calmly, care for myself better’ (P20, man in his 50s).

### 3.3 The Social Impact of VLDs: Renegotiating Roles, Ties and Identities

Illness reverberated far beyond the clinical sphere, affecting relationships, family roles, life plans, and work. Participants described disruptions in couple and parenting dynamics, constrained career paths, and financial strain, often compounded by the invisibility of symptoms and resulting misunderstanding. Overall, VLDs were experienced as reshaping social participation and personal trajectories.

#### 3.3.1 Relationships With Close and Extended Circles

##### 3.3.1.1 Gratitude and Guilt About Burdening Relatives

Partners and close relatives played a central role in daily support and crisis management, yet this constant vigilance often triggered feelings of burden and moral discomfort. One participant explained: ‘My family helps me a lot… though I feel bad because they're always on alert. They always have that worry, you know?’ (P2, woman in her 50s). To avoid adding to their relatives’ distress, several patients reported self‐censorship, choosing not to share ongoing fears or symptoms and striving to assert a ‘non‐sick’ version of themselves within the family sphere. As one woman noted, ‘Sometimes it's harder to say things to those close to you because they went through it with you… so you don't necessarily want to bring up old stories that they've put behind them’ (P11).

##### 3.3.1.2 Disbelief and Social Withdrawal From Extended Circles

In extended social circles, the invisibility of many symptoms created significant misunderstandings. Participants frequently encountered minimisation or disbelief, especially when physical appearance suggested recovery or even improvement: ‘Most people just say, ‘Well, you look fine’ because from the outside, you can't tell. I've even lost a lot of weight, so some think I look better’ (P6, man in his 50s). This discrepancy between visible normality and internal suffering contributed to feelings of illegitimacy and withdrawal. Repeated cancellations due to fatigue or medical issues strained friendships: ‘If you cancel four times, friends distance themselves’ (P24, man in his 50s). Over time, some participants reported selectively reducing social contacts to avoid explanations, judgement, or disappointment.

#### 3.3.2 Personal Life: Disrupted Life Projects and Expected Roles

Beyond immediate family relationships, the disease profoundly disrupted major social roles and life projects, particularly around reproduction, parenting, and couple relationships.

##### 3.3.2.1 Reshaping Reproductive Choices

For several women, the diagnosis confronted them with the painful reality that pregnancy was risky or medically contraindicated, reshaping reproductive choices and, in some cases, resulting in involuntary childlessness. Responses ranged from acceptance and reorientation—such as embracing childfree life or pursuing adoption—to attempts at pregnancy under strict medical surveillance, sometimes ending in repeated losses. One participant recounted: ‘I decided to take the risk [to have a child] — twice — and I lost both’ (P1, woman in her 50s).

##### 3.3.2.2 Diminished Capacity to Fulfil Expected Parental Roles

Parenthood, when possible, required constant adjustment to fluctuating energy levels and physical limitations. Chronic fatigue constrained everyday caregiving and participation in family activities, generating frustration and guilt: ‘Sometimes I can do things with my children, but other times I have so little energy that I just can't’ (P27, woman in her 30s). While women more frequently articulated the burden of reconciling illness and parental responsibilities, some men also expressed concern about their diminished capacity to fulfil expected parental roles.

##### 3.3.2.3 Challenging Couple Stability

Conjugal life was similarly affected by fatigue, dependence, and the redistribution of domestic and emotional labour. Participants described mismatched rhythms, altered intimacy, and tensions arising from unequal capacities, sometimes leading to relational strain or rupture: ‘It's true that from time to time, she gets a bit tired of my condition’ (P13, man in his 50s). These dynamics challenged not only couple stability but also participants’ sense of desirability, reciprocity, and moral worth within intimate relationships.

#### 3.3.3 Professional Trajectories: Constrained Careers and Concealed Vulnerability

##### 3.3.3.1 Feeling Limited in One's Career

Professional lives were also profoundly affected. Two participants were no longer able to work due to disease complications and relied on disability pensions, while many others reported reduced work capacity with lasting consequences for career progression and income. Several described accepting positions below their qualifications in order to accommodate fluctuating health: ‘I know that I have the intellectual level for a higher salary, but that also means you always have to be ‘on’. And I can't always be ‘on’, so I have to accept that… and therefore earn a lower salary’ (P24, man in his 50s).

The timing of diagnosis proved crucial: for those in mid‐life, professional interruption was experienced as particularly distressing, as it occurred at a moment when peers were consolidating careers and long‐term projects. As one woman explained: ‘It's different to get this at 40 than at 60… now it's frustrating because all my friends are working, and I can't even study—with hospitalisations and medical visits, I can't commit to any qualification program’ (P8, woman in her 40s).

##### 3.3.3.2 Fear/Experience of Discrimination at Work

Workplace disclosure was discussed as a sensitive, strategic choice. Fear of stigma and misunderstanding often led patients to conceal their condition, especially when symptoms like fatigue remained invisible. Managing illness privately required constant effort to maintain normal productivity: ‘I went back to work… they didn't understand, in the sense that it's not something visible from the outside… so I didn't tell them, I manage my fatigue as best as I can’ (P17, woman in her 50s). Others avoided disclosing to colleagues or occupational physicians: ‘It's something I never talked about professionally… we don't want to be stigmatised’ (P12). This concealment increased emotional strain and feelings of professional precariousness.

#### 3.3.4 The Social Cost of Illness

Beyond employment, participants described a broader ‘social cost’ of chronic illness, including out‐of‐pocket medical expenses (mainly for transportation, and, in Spain, for a type of medication), repeated insurance refusals, and the exhaustion associated with navigating complex administrative systems. These barriers intensified feelings of vulnerability and social marginalisation and fostered institutional mistrust and a sense of being treated as risky within bureaucratic systems. One participant explained: ‘Obtaining life and extended health insurance has been complicated… it always has to be made so difficult. I get tired of those organisations — you have to explain everything, and then they still don't understand’ (P25, woman in her 50s).

### 3.4 Healthcare Experience: From Relational to Institutional Gaps

Positive healthcare experiences were shaped by clear communication, continuity of care, and recognition of patients’ lived experiences, while minimisation of symptoms and side effects undermined trust. Small relational gestures were often remembered as meaningful signs of respect and recognition: ‘The effort made by the doctor to come and sit next to me at eye level meant a lot to me’ (P24, man in his 50s). By contrast, the minimisation or banalization of symptoms and treatment side effects was a recurrent source of frustration and sometimes led to tensions around therapeutic choices. One participant, whose quality of life deteriorated severely under interferon treatment, described deciding to discontinue therapy despite medical recommendations: ‘Better [to live] 10 good years than 20 with side effects’ (P10).

Beyond individual encounters, fragmented care, lack of psychological support, and insufficient attention to caregivers forced patients to coordinate their own care, illustrating how institutional gaps shape both practical and emotional aspects of living with VLDs. Participants pointed to systemic shortcomings in care coordination across services and institutions, reporting having to manage their own medical files, ensure information transfer between specialists, and organise follow‐up themselves. The absence of structured post‐hospitalisation follow‐up was sometimes experienced as a form of abandonment: ‘No aftercare, no conversation with a nurse who had treated me… I had to figure it all out myself’ (P26, man in his 50s).

### 3.5 The Sense of Belonging to a Community of Peers

Across all stages of the illness trajectory, participants emphasised the importance of connecting with peers living with the same condition as a potential resource, though one that is currently lacking. Peer support was valued both as a source of practical knowledge, helping patients navigate healthcare systems and manage symptoms, and as a space for exchanging experiential strategies for everyday adaptation, playing a crucial emotional and social role: ‘I'd like to meet other patients, even just for coffee. You don't have to talk about the illness all the time, but sometimes something comes up, and you realise someone else has the same little problem as you, and it's comforting’ (P2, woman in her 50's). Given the rarity of VLDs and the invisibility of many symptoms, contact with others facing similar challenges offered recognition, validation, and a sense of belonging, but only a few patients had the occasion to meet others, and thus appreciated the fact that they were interviewed by a PR.

## 4 Discussion

### 4.1 Brief Summary of Findings

By adopting qualitative participatory methods embedding patients’ voices at the core of the research design, this study provides an in‐depth understanding of how VLDs affect quality of life in everyday life contexts. Our study reveals patients’ perspectives, including the continuity of identity and social roles, social participation, acknowledgement of their illness, autonomy in daily life and treatment pathways, and the ability to live ‘as normally as possible’. These findings highlight quality of life in VLDs as a dynamic, biographical process shaped by social relationships, work trajectories, and long‐term uncertainty, rather than solely by clinical status and health‐related measures highlighting fatigue or depressive symptoms [11, 13].

### 4.2 Interpretation in Light of Previous Literature

Although HRQoL is commonly conceptualised as a multidimensional construct encompassing physical, psychological, and social domains, standard HRQoL instruments primarily capture symptom intensity and discrete life domains at specific time points, with poorer scores correlating with disease progression, intensity of complications and lower functional status [19]. However, these instruments tend to present HRQoL as a relatively static outcome and insufficiently capture the temporal dynamics, uncertainty, and adaptive processes that characterise long‐term trajectories in chronic diseases such as VLDs [11], as highlighted by our qualitative findings.

#### 4.2.1 Managing Uncertainty: Fatigue and the Need for Constant Anticipation

Prior research on chronic liver diseases has identified fatigue as one of the most frequently reported and debilitating symptoms, affecting physical performance, cognitive function, and social participation, across a wide spectrum of liver conditions [20]. Our findings highlight that, in VLDs, fatigue is not merely a matter of intensity but is experienced as unpredictable and fluctuating, requiring patients to constantly anticipate and plan everyday activities, such as caring for children, around uncertain energy levels. This ongoing need for anticipation makes fatigue profoundly disruptive, interfering with daily routines, work, and social engagement, which is in line with previous research in rare liver diseases [21]. Beyond these day‐to‐day effects, our study highlights anticipatory uncertainty in daily life as a major source of distress, reflecting the ongoing negotiation patients must engage in to manage their health and participation in social life.

Prior studies in chronic liver disease have linked complications such as ascites, recurrent hospitalisations, and hepatic encephalopathy to significant declines in both physical and mental HRQoL [9, 22, 23]. Our findings extend this knowledge by showing that acute episodes, such as hepatic encephalopathy, are experienced by patients as crises with potentially traumatic consequences, leaving lasting emotional effects. Beyond their acute physiological impact, these episodes also affect patients’ self‐image and sense of control, as individuals confront visible or perceived impairments in competence, autonomy, and social roles. Our findings indicate that the burden of disease is both cumulative and dynamic, requiring continuous adaptation to shifting bodily capacities, unpredictable crises, and their social and psychological consequences, as in many other chronic diseases [11, 24].

#### 4.2.2 Stigma, Social Identity, and the Invisibility of Rare Disease

Importantly, HRQoL frameworks often overlook structural and social dimensions of living with chronic and stigmatised conditions [10]. Strategies of concealment and fear of work discrimination mirror findings in other chronic and rare diseases [25]. Besides, for patients with VLDs, visible manifestations such as ascites and invisible symptoms like chronic fatigue and pain expose them to moralising social judgements (e.g., perceived as alcohol‐related; hypochondriacal), which can undermine social identity, exacerbate withdrawal, and contribute to isolation. This stigma is compounded by limited public and professional awareness, which may further delegitimize their experiences [21] and intensify isolation [26]. These dynamics reflect forms of epistemic injustice in healthcare, in which patients’ experiential knowledge is discounted due to stigma and limited disease‐specific expertise, leading to delayed recognition of symptoms and inadequate supportive care [27]. Peer support facilitating practical self‐management, as found in studies on cirrhosis and other chronic or rare conditions [28, 29, 30], may be a key coping resource.

#### 4.2.3 Beyond Functional Loss: Resilience, Self‐Management and Identity Preservation

Focusing solely on deficits may overshadow patients’ adaptive capacities. While HRQoL is traditionally measured through symptom severity and functional loss, participants emphasised resilience and positive aspects, echoing prior work in rare diseases [3, 31], but also highlighting the need for the preservation of identity and efforts made to maintain meaningful roles despite ongoing limitations [31, 32], such as treatment negotiation or discontinuation, or the choice of ‘dangerous’ physical activities.

However, prior work has highlighted that self‐management and everyday adaptations are embedded within social and material contexts [13, 33], reflecting broader tensions between autonomy and vulnerability which are probably amplified in rare liver diseases due to limited disease‐specific guidance and fragmented care pathways. Such nuances remain largely invisible in studies on HRQoL, yet they are central to understanding patient priorities and designing interventions that are socially meaningful, and responsive to lived experience.

### 4.3 Strengths and Limitations

To our knowledge, this study is the first qualitative investigation of HRQoL in patients with VLDs. Its inductive and participatory design is a major strength, allowing PRs to define what matters beyond predefined HRQoL dimensions to generate concrete, actionable insights. Involvement of patients and caregivers throughout the research process strengthened relevance and interpretive depth, while the international setting and limited country‐specific findings support transferability across contexts.

Several limitations should nevertheless be acknowledged. Although the sample size (27 interviews) is consistent with qualitative standards [34], data collection and analysis were not fully iterative, as guides and inclusion criteria were defined a priori, with a focus on descriptive rather than theoretical saturation. Furthermore, given the exploratory nature of the research question and the importance of maintaining a collaborative and equitable process, we chose to conduct a semantic thematic analysis using rapid qualitative techniques, which may have reduced analytical depth and complexity.

The participatory design of the study also raised methodological challenges. These included variability in interviewing and analytic skills between PRs, difficulties in discussing sensitive topics despite training, and differences in the availability and diversity of PRs throughout the project. Sustaining long‐term involvement was also challenging due to professional commitments, health issues, and emotional resonance with participants’ experiences. Representativeness of PRs in terms of age and gender was limited during the international workshop and subsequent analysis phases. However, the initial analyses conducted individually, with the analysis sheets, and collectively, during local workshops) were the most important ones for PRs to share their perspectives. Moreover, the iterative analytical process implemented during the second phase enabled half of the PRs to confirm the relevance and accuracy of the interpretations, suggest clarifications and refinements, and ensure that the final analytical framework adequately reflected perspectives across participating countries. These methodological and ethical issues, together with lessons learned from the project, are discussed further elsewhere [35].

Selection bias is possible due to recruitment via patient organisations and specialised clinical networks. In addition, some accounts reflect earlier treatment standards (e.g., pregnancy is not systematically discouraged as it used to be), limiting transferability to current clinical practice. Finally, healthcare systems were different, with three countries following the Bismarck model, and one (SP) following the Beveridge model. These structural differences likely contributed to variations in out of pocket‐expenses, as highlighted by the social cost of illness, which appeared to be distinct in Spain.

### 4.4 Practice Implications

Our findings highlight several domains that require systematic integration into routine hepatology care.

#### 4.4.1 Fatigue Management and Daily Functioning

Although non‐pharmacological interventions for fatigue and functional capacity (e.g., physical activity, psychosocial interventions) have demonstrated benefits, they remain underutilized in routine care [36]. Beyond symptom burden, patients struggle with uncertainty regarding safe activity levels, notably due to the risk of bleeding with anticoagulants in several activities. Given limited pharmacological options, structured counselling on energy management in everyday routines, pacing strategies, and adapted physical activity should be incorporated into routine follow‐up.

#### 4.4.2 Nutrition and Context‐Sensitive Lifestyle Support

Although nutritional interventions can reduce disease burden, their effectiveness depends on feasibility within patients’ social and economic contexts. Standardised dietary advice is often insufficient when work schedules, family meals, or symptom fluctuations are not considered. Dietetic counselling should therefore prioritise co‐constructed, context‐adapted strategies that support both disease management and socioeconomic context.

#### 4.4.3 Psychological Burden and Caregiver Support

Psychological distress related to chronic uncertainty and fear of deterioration is widely reported in chronic and rare diseases [12]. Our findings further show that symptom unpredictability and cognitive late effects affect family dynamics and caregiving roles, consistent with high caregiver burden in advanced liver disease [37, 38]. Although psychosocial interventions improve coping and functional outcomes [39, 40], access remains uneven in rare liver diseases. Routine psychological screening and clear referral pathways for both patients and relatives are therefore needed.

#### 4.4.4 Intimacy, Sexuality, and Reproductive Counselling

Disruptions in sexual and intimate life linked to fatigue, medication effects, anxiety, and body image concerns are reported in liver diseases but under‐addressed by healthcare providers [41, 42]. Women's accounts of constrained reproductive choices echo the call for individualised reproductive counselling in rare liver disease that remains inconsistently implemented [21]. Proactive, multidisciplinary counselling should be integrated into standard care pathways rather than addressed only when complications arise.

#### 4.4.5 Work, Financial Security, and Stigma

Reduced work ability, financial strain and barriers to social benefits align with evidence on the impact of rare diseases on employment [43] and the emerging concept of ‘financial toxicity’ in chronic liver disease [44]. Early referral to social workers for workplace accommodations, disability benefits, and insurance navigation could mitigate long‐term socioeconomic consequences [45], while broader public and policy interventions are needed to reduce stigma [46].

#### 4.4.6 Integrating Peer Support Into Care Pathways

Structured peer‐support programs remain uncommon in rare liver diseases. Integrating peer mentoring into clinical pathways, in collaboration with patient organisations, could reduce isolation and complement professional care, and clinicians should actively refer patients to these resources [47].

## 5 Conclusion

This study captured how individuals living with VLDs understand their quality of life and identify their unmet needs. By involving PRs across design, data collection, and analysis, the findings reflect patients’ perspectives and provide experience‐based insights to inform patient‐centred care. Living with a rare VLD reshapes everyday life, marked by uncertainty, fatigue, stigma, and social disruption, alongside efforts to preserve dignity and autonomy. While resilience often arises through adaptation, it should not be mistaken for the absence of need. Improving quality of life requires moving beyond biomedical outcomes to address psychological, social, occupational, and financial challenges, including barriers such as workplace exclusion or reproductive concerns. A patient‐centred approach therefore requires coordinated care, personalised guidance, recognition of caregivers, and support for social and professional participation. Ultimately, the goal is not only longer survival but meaningful living, which depends on close collaboration with patient organisations focused on rare liver diseases.

## Author Contributions

**Anouck Alary:** writing – original draft, visualisation, formal analysis, writing – review and editing. **Jane Sattoe:** conceptualisation, methodology, formal analysis, writing – review and editing, visualisation. **Lynda Sifer:** visualisation, formal analysis, writing – review and editing, methodology. **Isabelle Aujoulat:** conceptualisation, formal analysis, methodology, visualisation, writing – review and editing, funding acquisition. **José Mira:** methodology, conceptualisation, formal analysis, writing – review and editing, visualisation, funding acquisition. **Guenda Bernegger:** methodology, formal analysis, visualisation, writing – review and editing. **Giada Danesi:** methodology, formal analysis, visualisation, writing – review and editing. **Eva Gil‐Hernández:** methodology, formal analysis, visualisation, writing – review and editing. **Pura Ballester:** methodology, formal analysis, visualisation, writing – review and editing. **Mercedes Guilabert:** methodology, formal analysis, visualisation, writing – review and editing. **Doortje Nipius:** formal analysis, investigation, writing – review and editing. **Jeanina Kousemaker‐Nieuwdorp:** formal analysis, writing – review and editing, investigation. **Kay van der Meer‐Kuiper:** investigation, writing – review and editing, formal analysis. **Stefania Cannavò:** investigation, writing – review and editing, formal analysis. **Cecile Drossart:** investigation, writing – review and editing, formal analysis. **Liliane Veniat:** investigation, writing – review and editing, formal analysis. **María Caiata Zufferey:** conceptualisation, methodology, formal analysis, writing – review and editing, visualisation. **Agnes Dumas:** methodology, conceptualisation, formal analysis, data curation, writing – original draft, writing – review and editing, visualisation, supervision, project administration, funding acquisition.

## Conflicts of Interest

The authors declare no conflicts of interest.

## Data Availability

The data that support the findings of this study are available from the corresponding author upon reasonable request.
